# Astley Cooper Prize

**Published:** 1853-08

**Authors:** 


*Astley Cooper Prize.*—The fifth triennial prize of three hundred pounds, under the will of the late Sir Astley Cooper, Bart., will be awarded to the author of the best essay or treatise "*On the cause of the Coagulation of the Blood.*" The conditions are, that the essays or treatises written for such prize, shall contain original experiments and observations, not previously published, that they shall be illustrated by preparations and drawings as far the subject will admit, and that the successful essay become the exclusive property of Guy's Hospital. With the exception of the physicians, surgeons, or other officers for the time being, of Guy's or St. Thomas' Hospitals, and their relatives by blood or affinity, the prize is open for competition to the whole world. Essays intended for the prize must be sent in the usual manner, to the address of the physicians and surgeons of Guy's Hospital, on or before January 1, 1856.

